# Half-life modeling of basic fibroblast growth factor released from growth factor-eluting polyelectrolyte multilayers

**DOI:** 10.1038/s41598-021-89229-w

**Published:** 2021-05-07

**Authors:** Ivan Ding, Amy M. Peterson

**Affiliations:** 1 Department of Chemical Engineering, University of Massachusetts Lowell, One University Ave, Lowell, MA 01854 USA; 2 Department of Plastics Engineering, University of Massachusetts Lowell, One University Ave, Lowell, MA 01854 USA

**Keywords:** Biomaterials, Protein delivery, Biomaterials

## Abstract

Growth factor-eluting polymer systems have been widely reported to improve cell and tissue outcomes; however, measurements of actual growth factor concentration in cell culture conditions are limited. The problem is compounded by a lack of knowledge of growth factor half-lives, which impedes efforts to determine real-time growth factor concentrations. In this work, the half-life of basic fibroblast growth factor (FGF2) was determined using enzyme linked immunosorbent assay (ELISA). FGF2 release from polyelectrolyte multilayers (PEMs) was measured and the data was fit to a simple degradation model, allowing for the determination of FGF2 concentrations between 2 and 4 days of culture time. After the first hour, the FGF2 concentration for PEMs assembled at pH = 4 ranged from 2.67 ng/mL to 5.76 ng/mL, while for PEMs assembled at pH = 5, the concentration ranged from 0.62 ng/mL to 2.12 ng/mL. CRL-2352 fibroblasts were cultured on PEMs assembled at pH = 4 and pH = 5. After 2 days, the FGF2-eluting PEM conditions showed improved cell count and spreading. After 4 days, only the pH = 4 assembly condition had higher cells counts, while the PEM assembled at pH = 5 and PEM with no FGF2 showed increased spreading. Overall, the half-life model and cell culture study provide optimal concentration ranges for fibroblast proliferation and a framework for understanding how temporal FGF2 concentration may affect other cell types.

## Introduction

The controlled release of biologically relevant molecules is of great interest in the biomedical field, where exogenous methods of supplementation can be costly and risk contamination of products meant to directly treat patients^1^. Polyelectrolytes have been employed as core components of many controlled-release systems due to their versatility^2^. From the same starting materials, polyelectrolytes can form a number of different structures, including polyelectrolyte multilayer (PEM) coatings, polyelectrolyte complexes (PECs), complex coacervates, or microcapsules^3–12^. The structure and properties of the resulting material depend on factors including the assembly method, polyelectrolyte concentrations, pH, and temperature^3,13–15^. For this reason, polyelectrolytes have been used for a range of cell scaffolds and surface coatings capable of releasing specific nanoparticles, pharmaceuticals, and proteins^7–11,16^.

PEMs specifically have been used extensively to control the release of growth factors (GFs) such as basic fibroblast growth factor (FGF2), bone morphogenetic protein (BMP-2), and transforming growth factor beta 1 (TGF-β1)^3,6,17,18^. FGF2 is especially important for the growth of both fibroblasts and mesenchymal stem cells and is linked to improved proliferation, decreased cell death rate, and can also prevent differentiation to undesirable phenotypes^3,19–23^. PEMs are well-suited to the controlled release of GFs due to their ability to maintain GF bioactivity and release GFs over periods ranging from weeks to months^3,6,17^. Furthermore, when PEMs without GFs are used alone to modify culture surface properties, they can improve cell proliferation compared to common culture substrates and implanted device materials such as tissue culture plastic (TCP) and titanium^3,6,17^. The ability to simultaneously control surface properties and GF release rates, coupled with the ease of processing, make PEMs excellent materials for improving cell outcomes both in vitro and in vivo when paired with GFs.

While numerous publications report that GF-eluting PEMs greatly improve cell outcomes, many do not quantify the actual concentrations of GF released into the system^6,18,24,25^. Methods for measuring GF concentration include enzyme-linked immunosorbent assay (ELISA), fluorescent labeling, circular dichroism, and western blot^3–5,17,26–29^. However, all of these methods have limitations. Fluorescent labeling requires modification of the starting protein, potentially changing the properties of or damaging the GF of interest^30^. Circular dichroism and western blot require much higher concentrations than the 0.1–100 ng/mL optimal dosage of GFs such as FGF2 and the number of conditions that can be characterized simultaneously is limited by gel and sample counts^19,31–33^. ELISA may detect non-bioactive protein that possesses the necessary binding groups, and may not detect bioactive forms of the protein if they cannot bind with ELISA antibodies^34^. For both methods, these limitations are compounded by the fact that GFs degrade over time under physiological conditions. At a specific time point, the concentration detected will inevitably be lower than the true amount released due to the loss of GF bioactivity. Despite the impact of protein degradation on measurement accuracy, there is almost no research on the half-lives of even the most common growth factors. For FGF2, the half-life has been determined in the literature through western blot (4.7–13.7 h depending on media composition) and circular dichroism (6.3 h for native form FGF2)^31^. Han et al. measured FGF2 degradation in phosphate buffered saline (PBS) using ELISA, but no further analysis of the half-life was performed in their work^11^. Due to differences in predicted half-life depending on methodology, the half-life values collected from other methodologies cannot be used to specifically model the controlled release of FGF2 at the ng/mL concentration range. Generating an accurate model of FGF2 degradation using ELISA-derived results necessitates determining its ELISA-detectable half-life.

In this work, we developed a working model of growth factor release from PEMs and subsequent degradation by obtaining an ELISA-detectable half-life of FGF2 and applying it to a new dataset. Half-life values were obtained by measuring concentrations of FGF2 dissolved in either PBS or Dulbecco’s modified Eagle medium (DMEM) over a period of 4 days. PEMs consisting of poly-l-histidine (PLH) and poly(methacrylic acid) (PMAA) were prepared at a range of assembly pH values to obtain a new data set for GF release. Combining these results provided an ELISA-detectable concentration profile of FGF2 from the GF-eluting PEMs. The ability of the PEMs to improve cell proliferation and attachment was determined using CRL-2352 fibroblasts.

## Results and discussion

### PEM half-life

**Figure 1:**
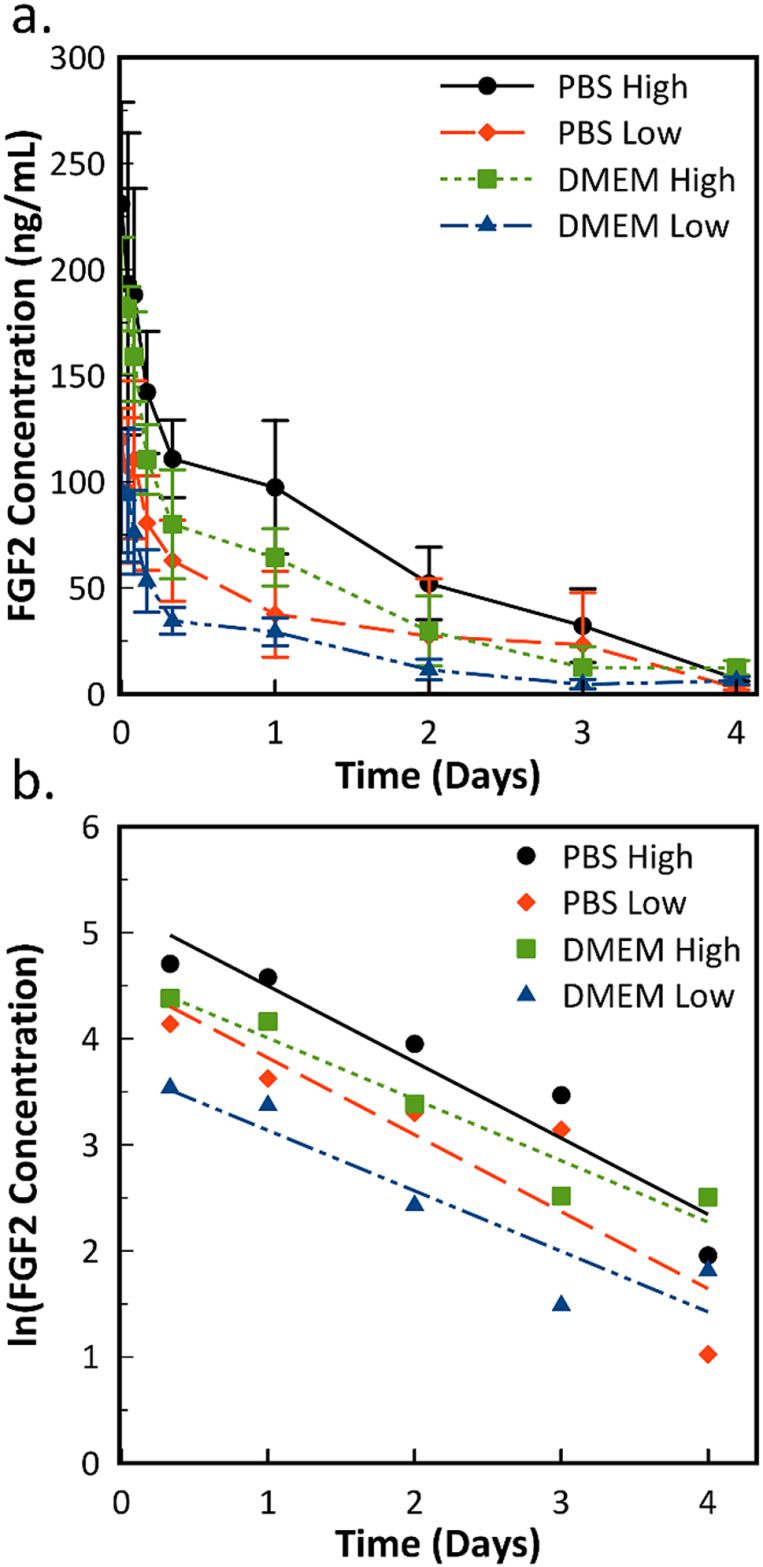
(**a**) ELISA-detectable FGF2 at high (500 ng/mL) and low (250 ng/mL) initial starting concentrations added exogenously to PBS and unmodified DMEM, measured over the course of 4 days. Error bars represent standard deviation (n = 5). (**b**) Natural logarithm of the same average concentration values with a linear fit from 8 to 96 h. The fit line is used to determine the degradation constant to calculate the half-life.

A degradation experiment was performed to determine the ELISA-detectable half-life for FGF2. We specify “ELISA-detectable” to differentiate the values from other methods that may result in different values. A high and low concentration of FGF2 (500 ng/mL and 250 ng/mL, respectively) were added directly to PBS or DMEM, and allowed to incubate at 37 °C. Aliquots were taken a specific time points to obtain a degradation curve. Figure 1 shows ELISA-detectable FGF2 concentration over the course of 4 days. The initial time point, taken 1 h after plating, shows a lower value than the expected starting concentration, likely due to a combination of a small amount of initial adsorption, including during mixing and transfer of aliquots to vials, and losses during freezing. Nonetheless, the results showed a gradual decrease in ELISA-detectable FGF2 concentration over the course of the 4-day study, resulting in an over tenfold drop in concentration for all conditions. For PBS, the final concentrations were 7.07 ± 0.97 ng/mL and 2.78 ± 0.94 ng/mL for high and low initial concentrations, respectively. For DMEM, the final concentrations were 12.25 ± 3.44 ng/mL and 6.13 ± 1.85 ng/mL for high and low, respectively. Final FGF2 concentrations in DMEM were statistically significantly higher than PBS conditions (p > 0.01).

Protein degradation has been studied using a two-state equilibrium model, where the protein in a native confirmation loses activity in an equilibrium reaction between folding/unfolding^29,35^. This model is described by Eq. (1).

1 $$ N\mathop \leftrightarrow \limits_{{}} U\to  F $$

*N* is the native state of a protein, *U* is the unfolded state, and *F* is any state where the protein can no longer return to the native state in any manner. When the first reaction (folding/unfolding) reaches equilibrium, the overall reaction can be treated as pseudo-first order. Thus, the degradation rate can be described by Eq. (2).

2 $$ \ln N\left( t \right) = \ln N_{0} - k*t $$

In Eq. (2), *t* is time, *N(t)* is the FGF2 concentration at that time, $N_{0}$ is the original concentration, and *k* is the first order rate constant describing the reaction of *U* to *F*. By taking the natural logarithm of the experimental concentration vs. time data and applying a linear fit, the k value can be obtained from the slope of the line.

FGF2 concentration vs. time data does not fit first order kinetics for the first four hours. This is likely because the folding/unfolding reaction does not reach equilibrium during this time. The FGF2 used for these experiments was reconstituted from a lyophilized state, diluted, then heated to the experimental temperature, so it is reasonable that equilibration would not be achieved immediately. The result is a fast degradation rate in the first few hours of the experiment. Half-life values based on all time points are provided in Table S.1, but are not used for modeling.

To account for the non-equilibrium conditions during initial plating, only data from 8 to 96 h was used in the calculation, as it more accurately describes long-term FGF2 degradation. Average half-life values were determined for each condition and are given in Table 1. No statistically significant differences were observed across experimental conditions. The lowest half-life is 22.50 ± 4.20 h and the highest is 29.82 ± 6.77 h. The results here indicate that FGF2 concentrations obtained in PBS-based release studies directly translate to conditions in cell culture media. Likewise, the consistency of half-life values between two different initial FGF2 concentrations indicates that the reduction over time in ELISA-detectable FGF2 is real and not due to long-term adsorption to the plate over the 4-day study. The half-life values in Table 1 are much higher than previously reported results using western blot and circular dichroism, which range from 4.7 to 13.7 h^31^. The data in the current work agrees better with the activity loss studied via ELISA presented in Han et al.^11^. This difference in reported values is likely a combination of both differences in detectable conformation of the proteins dependent on measurement method, and the difference between a short-term 24-h study compared to a 4-day study as it relates to protein equilibrium and early adsorption that occurs even to a blocked plate.

**Table 1:** Calculated FGF2 half-life values taken from ELISA data. Half-life values were obtained from individual curves for each replicate.

Condition	Half-life (h)	Std. dev. (h)	R^2^
PBS (500 ng/mL)	22.75	2.27	0.92
PBS (250 ng/mL)	22.50	4.20	0.81
DMEM (500 ng/mL)	27.95	3.11	0.94
DMEM (250 ng/mL)	29.82	6.77	0.85

### pH Dependence of PMAA/PLH PEMs

**Figure 2:**
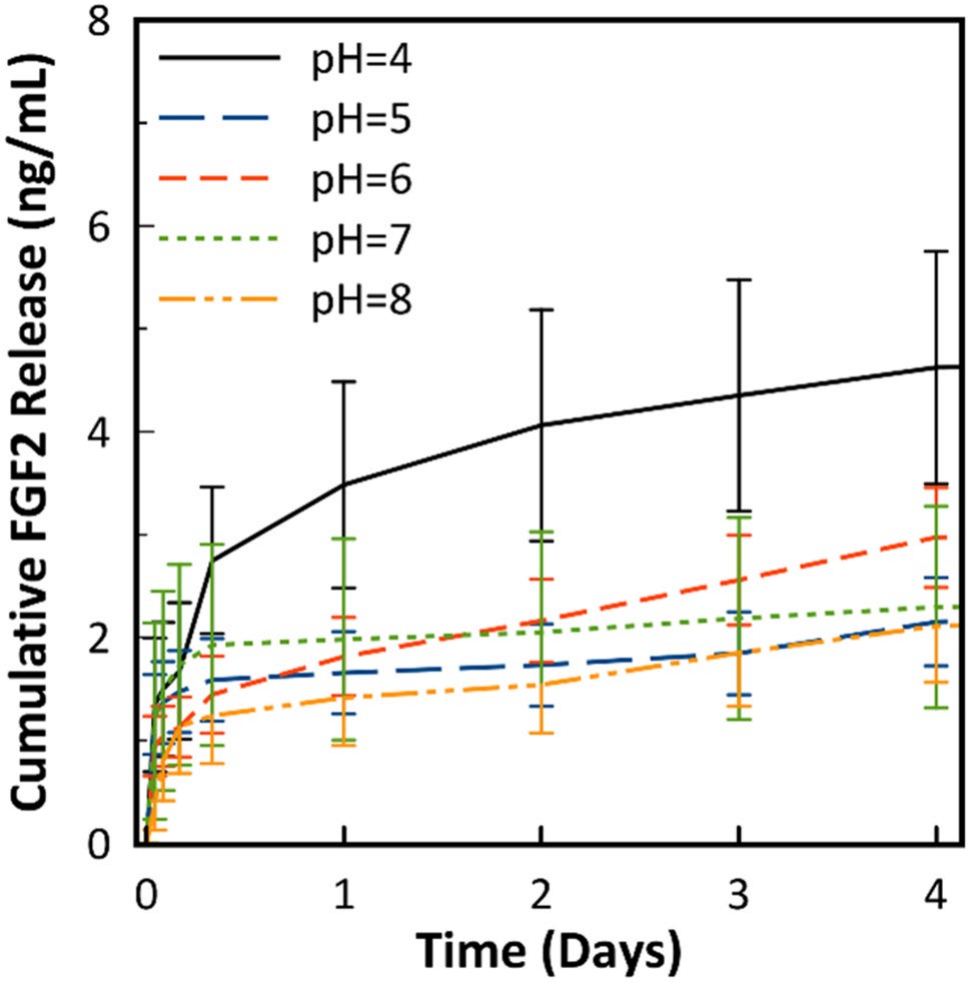
Cumulative FGF2 release over 4 days from FGF2-(PMAA/PLH)_5_ PEMs. Released data was obtained by immersing 1 cm^2^ substrates into 1 mL of PBS. 1 mL aliquots were taken and replaced over the course of 4 days. Error represents standard deviation (n = 5*).*

In order to both explore the effects of assembly pH on FGF2-eluting PEMs formed on tissue culture plastic, and to obtain a data set for modeling purposes, an FGF2 release study was performed on PEMs assembled at pH = 4, pH = 5, pH = 6, pH = 7, and pH = 8. 50 μg/mL FGF2 at pH = 7.4 was pipetted onto the substrate as the first layer as described in the Methods section. Figure 2 shows the cumulative release of FGF2 over 4 days of the release study. Total FGF2 released from the substrate can be seen in Supporting Information Figure S.1. Assembling the PEM at pH = 4 results in the highest FGF2 concentration after 7 days at 4.71 ± 1.13 ng/mL, which is statistically significantly higher than PEMs assembled at pH = 5, pH = 7, and pH = 8 (p < 0.05). FGF2 release from PEMs assembled at pH = 5 (2.3 ± 0.44 ng/mL) and above were not statistically significantly different.

**Figure 3:**
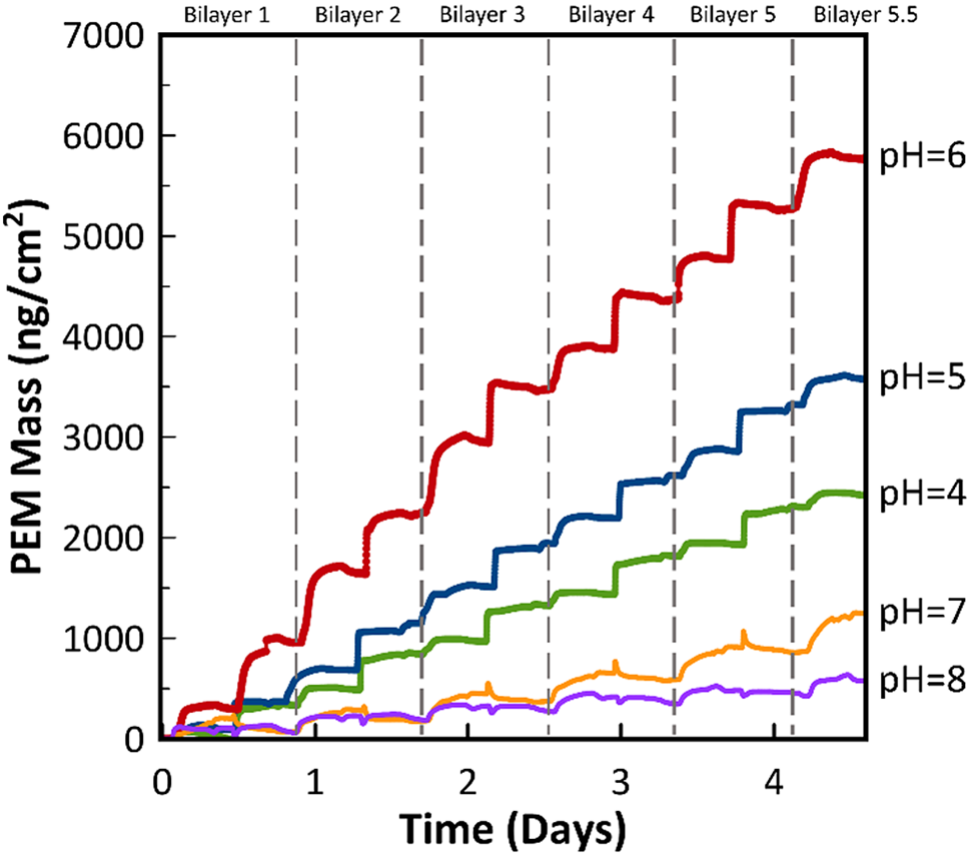
PEM mass during assembly obtained from QCM-D results for (PLH/PMAA)_5.5_ PEMs assembled at a pH range from pH = 4 to pH = 8. Polyelectrolyte and wash solutions were fed into the QCM-D at 50 μL/min for 15 min and 10 min, respectively. The final wash continued for an additional 10 min to observe any changes to the PEM.

Quartz crystal microbalance with dissipation monitoring (QCM-D) was performed to investigate how assembly pH affects PEM structure because the underlying structure of the PEM can affect FGF2 release^3^. The results are shown in Fig. 3. From pH = 4 to pH = 6, the thickness of the PEM increased with increasing pH. Likewise, the ratio of PLH to PMAA in each bilayer increased. This trend only holds true until pH = 7, where there is a sharp drop in PEM mass. At pH = 7, PLH visibly begins to precipitate in the storage vial, forming what appears to be a complex coacervate, likely due to the mixture of positive and negative charges common for polyamino acids at neutral pH values. The QCM-D results show that there is minimal adsorption of the PMAA layer and PEM formation is primarily driven by PLH at pH = 7. At pH = 8, the addition of PMAA results in rapid stripping followed by adsorption resulting in a mass slightly below or equal to the initial value.

From pH = 4 to pH = 6, PEMs grow steadily with increasing mass at different rates for each pH value. Changes in PEM mass with assembly pH are expected for weak polyelectrolytes such as the ones used in this work. Similar results have been seen in the literature for poly(acrylic acid)/poly(allylamine hydrochloride) PEMs, albeit in a much smaller pH range^36^. For PMAA/PLH PEMs, it is likely that polyelectrolytes have the highest charge density at a pH values ranging from 5 to 6, resulting in a driving force favoring adsorption. In even the very first bilayer under these assembly conditions, we see greater PLH adsorption that is then balanced by a greater mass of PMAA adsorbed. This behavior is repeated throughout the adsorption process, resulting in pH = 6 PEMs having twice the mass of pH = 4 PEMs.

Similarly, the collapse of PEM mass in the neutral pH range occurs as the ionization of both individual polyelectrolytes decreases^36^. For PMAA and PLH, this leads to instability in the PEM resulting in stripping. During PEM assembly, adsorption and stripping directly compete^37^. On an individual layer level, it can be seen that stripping overtakes adsorption when PMAA is introduced at pH = 7 and pH = 8, resulting in an initial loss of polyelectrolyte mass before more is adsorbed in that same layer. As PEMs are dynamic, rearrange during formation, and do not typically form discrete layers, mass loss likely correlates to a simultaneous loss in FGF2.

By comparing QCM-D results to FGF2 release curves, some conclusions can be drawn. The pH = 7 and pH = 8 conditions exhibit initial stripping of the adsorbed PEM when a new layer is added, which is correlated to an overall lower FGF2 release. In turn, this indicates that stripping of the PEM also results in FGF2 loss. For the stable PEMs at the pH = 4 to pH = 6 range, increasing PEM mass is correlated to lower FGF2 release rates. A partial explanation is that a thicker PEM may reduce the FGF2 diffusion rate, but there is no trend observed. Overall FGF2 release for both pH = 5 and pH = 6 are statistically significantly different from pH = 4, but not from each other, indicating there is an additional mechanism controlling FGF2 release unrelated to mass. A possible explanation is that the PEMs at pH = 5 and pH = 6 conditions are less able to maintain FGF2 bioactivity compared to the pH = 4 condition. Differences in chain arrangement, charge, and moisture uptake in the internal structure of the PEM at the higher pH values may all result in decreased FGF2 bioactivity.

Comparing the remaining amount of FGF2 adsorbed to the substrate after the release study provides further insight on the differences of the assembly pH conditions (see Supporting Information Figure S.1). Remaining FGF2 surface concentrations were not statistically significantly different for the pH = 4, pH = 5, and pH = 6 samples (6.7 ± 2.31 ng/cm^2^, 6.6 ± 2.95 ng/cm^2^, 4.2 ± 0.51 ng/cm^2^ respectively), indicating that PEM mass may play a partial role in controlling FGF2 diffusion rates. However, the amount of remaining FGF2 for the pH = 7 and pH = 8 conditions are lower, at 3.1 ± 1.30 ng/cm^2^ and 0.6 ± 0.22 ng/cm^2^, respectively. This reinforces the idea that less FGF2 remained adsorbed in the pH = 7 and pH = 8 PEMs, but not at the lower assembly pH values. However, as the amounts of FGF2 released were statistically similar throughout the pH = 5 to pH = 8 range, it is clear that the release kinetics and arrangement of the polyelectrolyte chains within the PEM differ for each assembly pH condition.

PEM stability is likely the greatest contributing factor to the GF release results. Previous work on titanium-coated substrates showed that mass loss when exposed to PBS was minimized for a PEM formed at pH = 4, compared to those formed at pH values closer to that of PBS (pH = 7.4)^17^. In this system, polyelectrolyte choice plays an important role and the most stable PMAA/PLH PEM forms at pH = 4 where the positive charge of the polyamino acid, PLH, is greatest among the tested conditions. This increased stability results in a coating that is most able to maintain and release ELISA-detectable FGF2.

### Concentration modeling

A model of FGF2 solution concentration was developed by applying the ELISA-detectable FGF2 half-life value to the release study data. The model was generated iteratively in MATLAB, employing two simultaneous equations accounting for first order degradation Eq. (2) and growth factor release in the form of the power law model Eq. (3)^38^.

3 $$ \frac{{M_{t} }}{{M_{\infty } }} = Kt^{n} $$

*M*_*t*_ is the cumulative release up to a specific time *t*, *M*_*∞*_ is the total amount of drug released after an infinite time period, and *K* is a constant representing geometric factors of the release systems. *M*_*∞*_ was taken as the cumulative release plus the recovered FGF2 from an acid–base wash. *n* is a constant representing the mode of release^38,39^. Power law constants for each pH condition are provided in Table S.2. The low values of n seen in this work (n ≤ 0.315) show that release is not driven solely by a combination of Fickian diffusion and swelling, but provides no further information on the exact modes of release. Intermolecular bonding within PEMs includes ionic and hydrogen bonding, both of which are non-covalent, dynamic interactions. Release from PEMs is likely modulated by these types of intermolecular bonds. For complex systems, the power law is more accurate that other release models such as the Higuchi Equation, which assumes purely Fickian diffusion^38^.

**Figure 4:**
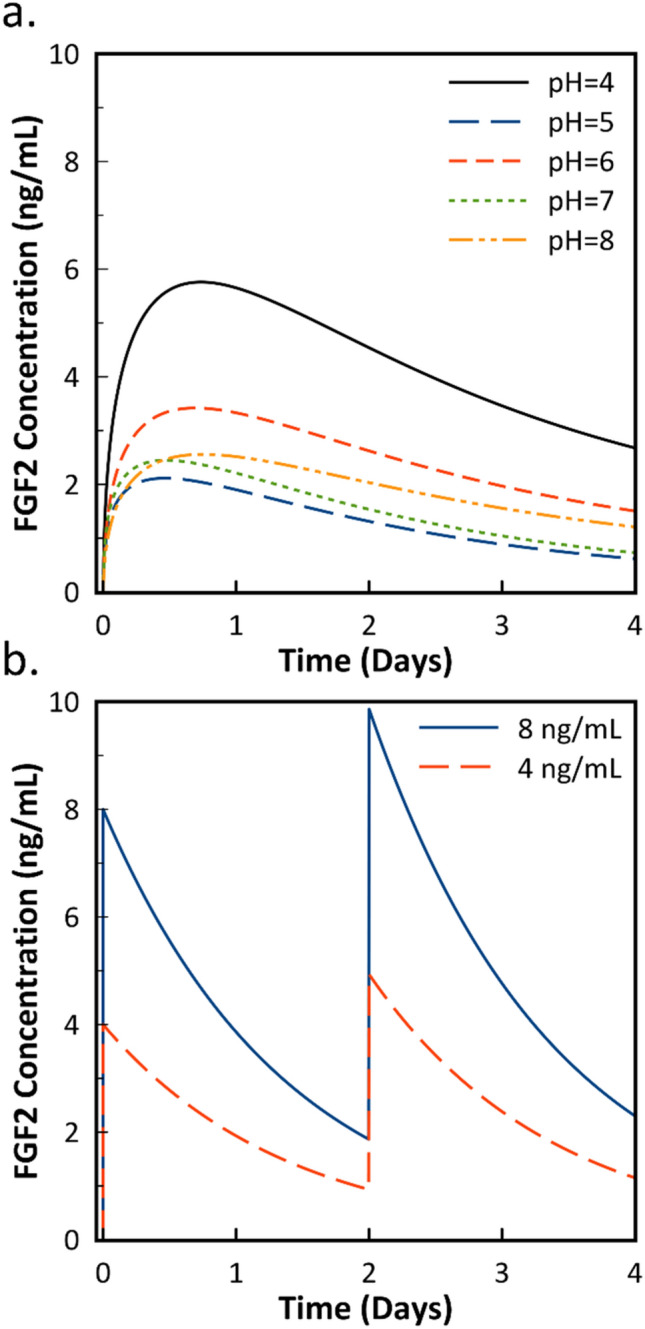
(**a**) Modeled concentration of FGF2 released from PEMs. pH values refer to PEM assembly conditions. Model assumes a 1.9 cm^2^ surface area and 600 μL of media. (**b**) Modeled FGF2 concentrations assuming exogenous supplementation of FGF2 at 4 ng/mL, and 8 ng/mL, at both t = 0 and t = 2 days. Both figures assume FGF2 degradation based on an ELISA-detectable half-life of 22.75 h.

Before fitting the experimental release data to the power law, the numerical values were adjusted using the experimentally obtained half-life value in order to account for degradation that occurs during the release studies themselves. The chosen half-life value was 22.75 h, which was taken from the higher concentration PBS data from Table 1 due to its lower relative standard deviation. After the adjusted release curve was obtained, Eqs. (2) and (3) could be solved numerically to obtain a concentration vs. time model that accounts for FGF2 degradation. Figure 4a shows the final graph produced using this methodology when applied to the data shown originally in Fig. 2. All values are adjusted for a 1.9 cm^2^ surface area and 600 μL of media. Intermediate graphs of calculated data are shown in the Supporting Information. Figure S.3 shows the raw release data fit to the power law, while Figure S.4 displays the release data accounting for surface area and volume differences in our cell culture system with and without degradation. The exogenous supplementation data seen in Fig. 4b was obtained by applying Eq. (2) by itself to an initial FGF2 media concentration of either 4 or 8 ng/mL. The exogenous supplementation conditions are representative of those commonly used for myoblasts and human dermal fibroblasts^40–43^.

FGF2 concentrations generally follow the trends seen in the release study, with the highest cumulative release resulting in the highest FGF2 concentration over time. FGF2 concentrations increase rapidly over the course of the first day, but gradually decline over the next 3 days. Figure 4b shows the FGF2 concentration curve for an exogenous condition where FGF2 is supplemented initially and after 2 days without total media replacement. For the exogenous condition to have the same amount of FGF2 after 4 days as the pH = 4 condition, an initial concentration between 8 and 16 ng/mL of FGF2 would be required. Table 2 shows the modeled FGF2 concentrations after 2 days and 4 days. When 16 ng/mL of exogenous FGF2 is added both initially and after 2 days, the FGF2 concentration range reaches as low as 3.71 ng/mL before supplementation and as high as 19.71 ng/mL after supplementation. For 8 ng/mL, the concentration decreases as low as 1.86 ng/mL. In order to maintain FGF2 concentrations through exogenous means, a more rigorous supplementation cycle is required, resulting in more physical interaction with the culture system, risking contamination. These results demonstrate a major benefit of GF-eluting PEMs compared to traditional methods.

**Table 2:** Modeled growth factor concentrations after 2 days and 4 days of culture time.

	PEM					Exogenous FGF2		
	pH = 4	pH = 5	pH = 6	pH = 7	pH = 8	16 ng/mL	8 ng/mL	4 ng/mL
2 day	4.53	1.31	2.61	1.53	2.03	3.72	1.86	0.93
4 day	2.67	0.62	1.49	0.73	1.20	4.58	2.28	1.14

For the exogenous FGF2 conditions, values were taken before additional FGF2 was supplemented.

Application of the short-term western blot-based values for FGF2 half-life, which range from 4.7 to 13.7 h, results in underestimation of ELISA-detectable FGF2 concentrations in the media^3,31^. Based on the presented model, it is clear that the ability of FGF-eluting PEMs to maintain FGF2 concentrations over time can be better modeled using an ELISA-detectable half-life.

### Cell culture

A cell culture study was performed with CRL-2352 fibroblasts to determine the performance of the GF-eluting PEM compared to exogenous FGF2 supplementation. This cell line was chosen because it is highly proliferative in the presence of FGF2^3,40,44^. Based on the release profiles obtained from the model, high and low FGF2-eluting PEMs (pH = 4 and pH = 5 respectively) were chosen as experimental conditions. Two exogenous conditions where 4 ng/mL and 8 ng/mL of FGF2 were added at t = 0 days and t = 2 days were chosen to bracket the lower and upper concentration ranges of the FGF2-eluting PEMs. The exogenous conditions had a PEM coating with no initial adsorbed FGF2 layer to act as a direct comparison to FGF-eluting surfaces. PEM-only and uncoated surfaces served as controls.

**Figure 5:**
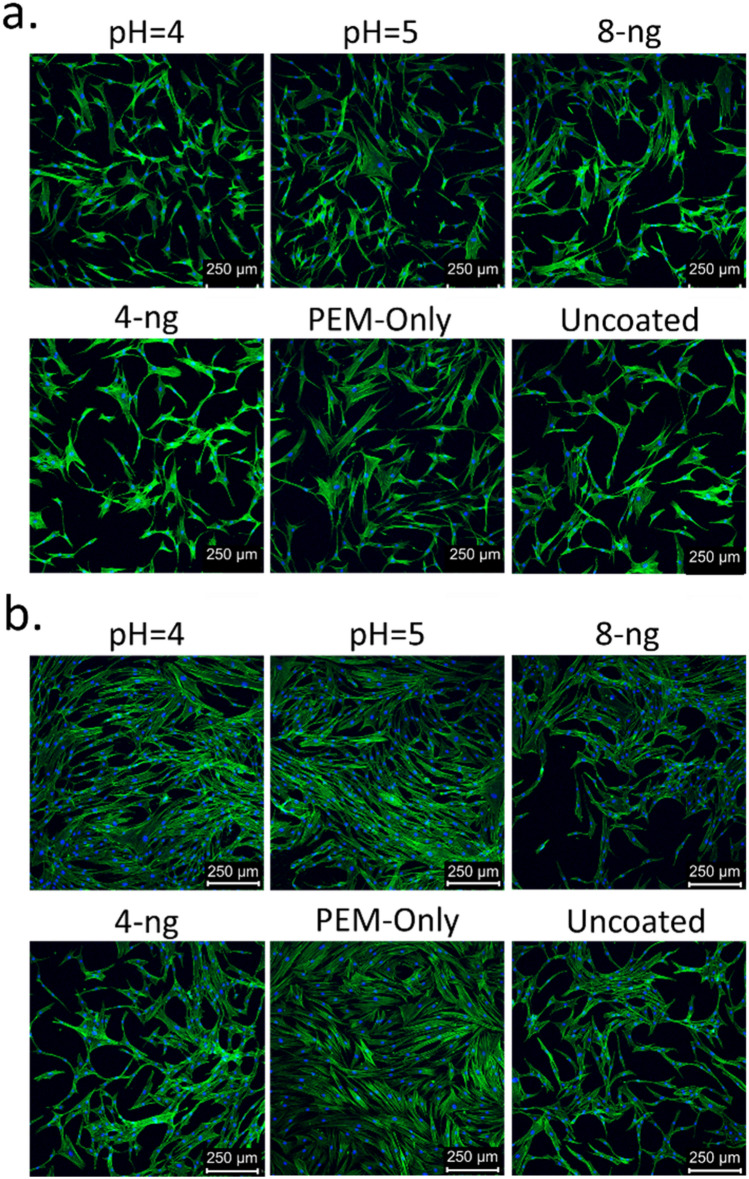
Representative images of cells stained with Hoechst 33342 (blue) and phalloidin coupled with Alexa Fluor 488 (green) at (**a**) 2 days and (**b**) 4 days of culture time. pH values refer to PEM assembly conditions. Cells were seeded at an initial density of 5000 cells/cm^2^ in IMDM with 1% FBS.

Figure 5 shows representative fluorescence images of cells labeled with Hoechst 33342 (blue) and phalloidin (green). At 2 days, the cells are morphologically similar across culture conditions. The actin cytoskeleton shows the cells are elongated in all conditions, indicative of a fibroblastic character^44^.

**Figure 6:**
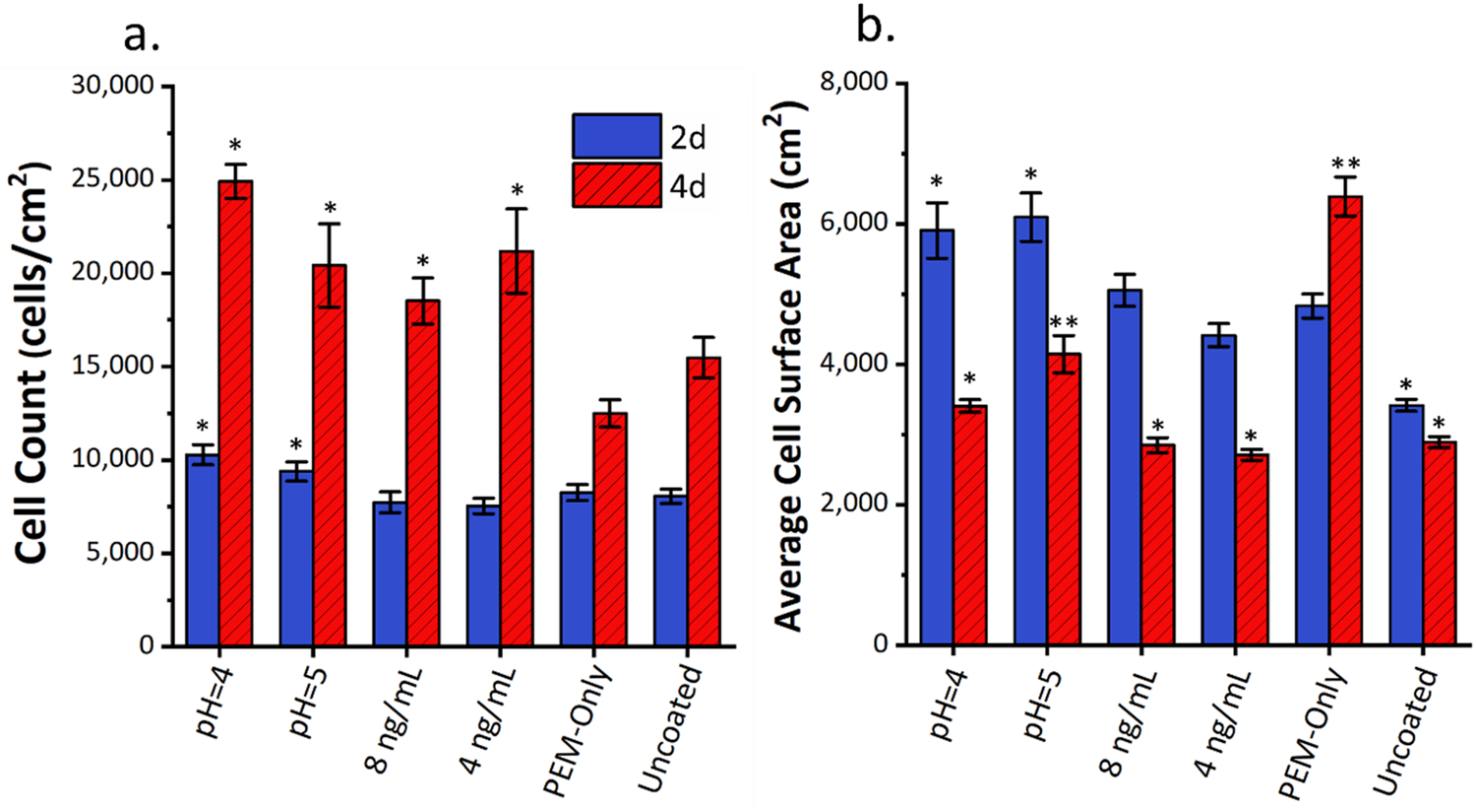
Comparison between 2-day and 4-day results for (**a**) cell counts obtained from Hoechst 33342 nuclear staining and (**b**) average cell surface areas from phalloidin labeling. Error bars represent standard error (n = 12). *Represents statistical differences from PEM-only control of the same time point (p < 0.05). **Represent statistical differences from all other conditions at the same time point (p < 0.05).

Figure 6a shows cell counts at the two time points. At 2 days, cell counts for pH = 4 and pH = 5 are 10,278 ± 1857 cells/cm^2^ and 9387 ± 1785 cells/cm^2^ respectively. These are statistically significantly higher than the other conditions, which are comparable to the uncoated control at 8056 ± 1322 cells/cm^2^. The cell counts are close to the seeding density, indicating that not all cells attached initially in low serum conditions. Figure 6b shows the average cell surface area. At 2 days, the pH = 4 (5905 ± 1568 μm^2^) and pH = 5 (6094 ± 1192 μm^2^) FGF2-eluting PEMs had higher average cell surface areas than all other conditions. The 8-ng condition (5052 ± 789 μm^2^) and PEM-only condition (4829 ± 594 μm^2^) have higher cell surface areas than the uncoated control (3414 ± 296 μm^2^). This indicates that the PEM coating by itself increases cell attachment and spreading after two days of culture time. However, only the FGF2-eluting PEM conditions resulted in increased cell counts at 2 days.

The results at 4 days do not follow the trends seen at 2 days. At 4 days, all FGF2-positive conditions show statistically significant increases in cell count compared to the PEM-only and uncoated controls. This is reasonable, as proliferation signaling is a primary function of FGF2^44,45^. Furthermore, the FGF2-eluting PEM at pH = 4 has a statistically significantly higher cell count than the 8-ng exogenous condition, indicating that the FGF2-eluting PEM may be superior to exogenous delivery at certain concentrations. However, for both GF-eluting PEMs and exogenous FGF2, control of concentration is extremely important.

Interestingly, the order of relative cell sizes differs greatly at 4 days compared to 2 days. At 4 days, the largest cells are found in the PEM-only condition, followed by pH = 5, then all other conditions. The PEM-only condition has a similar cell count to the uncoated control, but almost double the cell size. In part, the decreased cell spreading in the FGF2-positive conditions may be due to higher cell densities resulting in contact inhibition. However, this is likely a very small contributing factor. CRL-2352 fibroblasts do not purely form monolayers, and only reach 100% confluence at 40,000 cells/cm^2^. Furthermore, the pH = 4 condition has similar cell sizes to the 8 ng/mL condition despite differences in cell count. The fact that the cell size is second largest in the pH = 5 condition, the condition with the lowest FGF2 concentration, indicates that the PEM and FGF2 have competing effects on cell size. Based on the model, FGF2 concentrations in the pH = 5 condition remains below 2 ng/mL after the first day of culture and drops to as low as 0.62 ng/mL after 4 days. While the 4-ng exogenous condition reaches as low as 0.93 ng/mL at 2 days, there is a spike in concentration after supplementation at that time point. A sufficient FGF2 concentration likely reduces cell size due to the ability of FGF2 to maintain a more fibroblastic character, despite changes in morphology than can be induced by the PEM coating.

Both concentration and method of FGF2 presentation likely play a role in the proliferation results. When comparing the FGF2-eluting and exogenous conditions as a whole, FGF2 adsorbed into the PEM either improves cell adhesion or is more readily available to the cell upon initial attachment, causing an increased cell count at the 2-day mark. However, a specific concentration range may be necessary to optimize cell proliferation. Lower cell counts in the 8 ng/mL condition suggest that the FGF2 concentration is too high, reducing its effectiveness. On the other end of the spectrum, the FGF2 concentration achieved by the pH = 5 PEM is insufficient to achieve maximum cell proliferation. From 2 to 4 days, the pH = 5 condition underwent 2.18 population doublings, compared to the 2.4–2.8 population doublings seen in the other FGF2-containing conditions. As the pH = 5 condition has the lowest maximum FGF2 concentration at 2.11 ng/mL, which decreases to as low as 0.62 ng/mL at 4 days, there may simply be an insufficient amount of FGF2 in the system after the 2-day time point. Based on these results, optimal FGF2 concentration is in the range of 2.11–9.86 ng/mL.

Data for the cell surface areas is more difficult to interpret as the PEM and FGF2 simultaneously affect attachment. For the 2-day results, it is clear that FGF2-eluting PEMs improve either growth or initial attachment during seeding. This is likely due to the cells interacting with the FGF2 directly bound onto the surface. The PEM alone does not show this effect, nor does the PEM combined with exogenous FGF2. For that reason, we can conclude that presentation of FGF2 through direct contact with the PEM improves the viable number of cells during early culture over either material alone.

Care must be taken in modifying substrate properties—while improved fibroblast spreading is correlated with improved attachment, it may also be indicative of a more myofibroblastic, chondrogenic, or osteoblastic character^44,46^. However, this concern can be addressed by supplementing FGF2 exogenously, or releasing it over time when culturing cells on a PEM. FGF2 is known to allow fibroblasts to maintain their phenotype more effectively. Ideally, the PEM improves attachment by increasing the surface charge, while the FGF2 prevents undesired differentiation and improves proliferation. This could have significant implications for cell expansion in bioreactor systems, as the shear forces risk the causing detachment or undesired differentiation of cells^28,47^.

From a proliferation standpoint, the (PMAA/PLH)_5_ PEM and uncoated polystyrene have statistically similar cell counts, which is consistent with our previous findings^3^. However, confocal imaging and staining highlight that the extent of cell spreading is affected by the presence of a PEM. While exogenous addition of FGF2 to media is known to improve fibroblast proliferation^3,44,45^, this work provides a better understanding of the optimal concentration ranges under which FGF2 should be supplemented. Furthermore, FGF2-eluting PEMs can increase fibroblast growth rates, likely due to improvements in early attachment and growth.

## Conclusions

In this work, an ELISA-detectable half-life value was obtained for FGF2. This half-life was then used to model actual FGF2 concentrations in cell culture systems. While the half-life model developed in this work was applied to a specific PEM, the model can be applied to any culture environment where FGF2 vs. time data is available, including traditional culture systems and those containing biomaterials capable of controlled release. Likewise, the model can be expanded to other growth factor-based systems if an ELISA-detectable half-life is known. Since the model is based on ELISA-detectable concentrations of FGF2, the half-life of FGF2 was determined at concentrations that are relevant to culture of cells such as fibroblasts and myoblasts.

The effects of assembly pH on FGF2 release was studied and serves as a representative system for the developed model. The highest FGF2 release rate was seen from PEMs assembled at pH = 4, while PEMs assembled at pH = 5 to pH = 8 showed approximately equal FGF2 release rates. Based on QCM-D results, the differences in release profile can be attributed to the ability of the PEM at this pH to better maintain growth factor bioactivity, and not related to PEM mass directly. The model showed that the pH = 4 condition could maintain FGF2 concentrations between 2 and 6 ng/mL after 8 h of culture time in a cell culture system with a 1.9 cm^2^ surface area and 600 μL of cell culture media. The PEM assembled at pH = 5 could maintain a concentration between 0.5 and 2.5 ng/mL.

Fibroblasts were cultured at these same conditions to determine the effects of the FGF2-eluting PEMs on cell attachment and proliferation. After 2 days, the FGF2-eluting PEMs had increased cell counts and spreading compared to exogenous FGF2 supplementation and the FGF2-free controls. At 4 days, the cell count in the FGF2-eluting PEM assembled at pH = 4 was higher than both the 8 ng/mL exogenous condition and the controls. Cell spreading was highest in the PEM-only control followed by the FGF2-eluting PEM assembled at pH = 5, indicating that the PEM and FGF2 may have a synergistic effect on cell attachment and proliferation.

These results show that FGF2 introduced from a GF-eluting PEM is capable of moderately increasing the number of viable cells early during culture. It has been established that presentation of other GFs from the surface directly can provide more bioavailable GF. This work is an early sign that this also applies to FGF2 released from a PEM. However, the observed benefits are limited to the first 2 days of cell culture. Exogenous FGF2 and FGF2-eluting PEMs have an approximately equal number of population doublings between the 2-day and 4-day time points. In addition to the benefits during early culture time, usage of an FGF2-eluting PEM eliminates the need for multiple FGF2 supplementation steps, reducing human intervention and the associated risk of contamination and variability. The presence of a PEM improves cell spreading and attachment, which is important for higher shear environments such as bioreactors. Altogether, FGF2 eluting PEMs provide practical improvements to traditional cell culture techniques and substrates.

## Methods

### Materials

Plasma-treated polystyrene petri dishes were purchased from Corning. Poly(methacrylic acid) sodium salt (PMAA, 30 wt% solution, MW 4000–6000), poly-l-histidine hydrochloride (PLH, mol wt. ≥ 5000), PBS tablets (pH = 7.4), hydrochloric acid (12.1 M), and tris(hydroxymethyl)aminomethane were obtained from Sigma-Aldrich. Sodium hydroxide (10 N) and reagent grade ethyl alcohol was obtained from Fisher Scientific.

CRL-2352 fibroblasts were obtained from the American Type Culture Collection (ATCC). Gibco Iscove’s Modified Dulbecco’s Medium (IMDM) solution and Gibco fetal bovine serum (FBS) were obtained from Life Technologies Corporation. Penicillin streptomycin, 0.25%/2 mM trypsin–EDTA, Dulbecco’s Phosphate Buffered Saline (DPBS) and Alexa Fluor 488-coupled phalloidin were obtained from Thermo Fisher Scientific.

### FGF2 half-life determination

FGF2’s half-life was determined in both PBS and DMEM to understand its degradation in a simulated cell culture environment. No additives such as fetal bovine serum were included, as they vary in concentration depending on the culture system. Before the study, 5 wells in a 6-well polystyrene cell culture plate were blocked with 2% BSA overnight at 37 °C to limit FGF2 adsorption during the experiment. After blocking, the plates were washed with 37 °C PBS twice after which 4 mL of PBS or DMEM with the appropriate FGF2 concentration were added to the wells. At the appropriate time point, a 100 μL aliquot was taken from each of the wells without replacement and stored frozen until the ELISA study. The first time point was after a 1-h incubation period at 37 °C to limit the effects of adsorption to the well walls on the results. To account for non-equilibrium conditions, half-life results were obtained by fitting the data from 8 to 96 h to a first order rate law.

### Substrate preparation and PEM coating

Polyelectrolytes were prepared at a concentration of 1 mg/mL and Tris buffer was prepared at 0.05 M. Solution pH values were adjusted using 1 M HCl and 1 M NaOH. Before coating, the substrates were cut into 1 cm^2^ squares and cleaned with deionized water, ethanol, and deionized water again. Following the cleaning, 50 µg/mL FGF2 in pH = 7.6 Tris buffer was pipetted onto one side of the substrate and allowed to adsorb for 15 min, followed by three washes in deionized water for 1 min each. Using a dip coater (6 Position Compact SILAR Coating System, MTI), the substrates were immersed into 1 mg/mL PMAA for 15 min at the appropriate pH followed by two 90-s wash steps with water at the same pH. Another 15-min adsorption step was performed for 1 mg/mL PLH, followed by two more wash steps. These two steps were repeated five times to form 5 bilayers, resulting in a FGF2-(PMAA/PLH)_5_ PEM. The samples were dried using compressed nitrogen gas and stored at 4 °C overnight.

### FGF2 release study

To determine FGF2 release rates, the coated substrates were immersed in 1 mL of PBS in individual scintillation vials and incubated at 37 °C. At the specified time points, PBS was collected and stored frozen at − 30 °C and 1 mL of fresh PBS was added to the vial. After collecting the aliquots at the 7-day time point, substrates were rinsed for 30 s in 0.5 mL of 0.1 M HCl followed by a second rinse with 0.5 mL of 0.1 M NaOH. The HCl and NaOH aliquots were combined, pH adjusted to pH = 7.4 and stored at − 30 °C^4^.

ABTS sandwich ELISA kits (PeproTech) were used to quantify FGF2 release and ELISA was performed in accordance to supplier instructions. Aliquots from the release studies were thawed and returned to room temperature immediately prior to their use. Absorbance readings of the developed ELISA plates were obtained using a Molecular Devices SpectraMax M2 plate reader at a reading wavelength of 405 nm with a reference wavelength of 650 nm.

### Quartz crystal microbalance with dissipation monitoring

QCM-D was used to monitor PEM assembly at different pH values using a Q-Sense E4 (Biolin Scientific) system with polystyrene-coated sensors to mimic the experimental substrate. 1 mg/mL polyelectrolyte solutions were fed at a 50 µL/min flow rate for each layer followed by a 10-min wash step using water of the appropriate pH. An initial PLH first layer was used in place of FGF2 as both are positively charged below pH = 7. The final wash step continued for an addition 10 min to observe any changes to the PEM. Frequency and dissipation were obtained at odd overtones from the 3rd to 13th representing the harmonic resonances of the quartz crystal. The 3rd overtone was chosen to calculate the PEM mass as it best represents the bulk character of the film^48^. The Sauerbrey equation for rigid films was used to determine the mass of the assembled PEM based on the raw frequency data (Eq. 4):

4 $$ \Delta m = \frac{ - C}{{n\Delta f}} $$

where *m* is the calculated adsorbed mass, *f* is the resonant frequency, *n* is the overtone number, and *C* is the sensitivity constant, which is 17.7 ng/(cm^2^ Hz) for these 5 MHz QCM-D sensors. Use of the Sauerbrey equation is appropriate if the films are rigid, characterized by a stable ratio of dissipation change to frequency change between overtones and is generally applicable for any PEM system under 40 nm in thickness^49^.

### Cell culture

Individual 4-well plates were coated manually under the same assembly conditions as described above. In place of dip coating, the solutions were deposited using a multi-pipettor and were aspirated after the appropriate time interval. 200 µL of 50 µg/mL FGF2 was pipetted to the bottom of the well, followed by 300 µL of DI water, then by 300 µL of polyelectrolyte solution and pH-adjusted wash water for each step, forming the full PEM. GF-eluting PEMs were prepared at pH = 4 and pH = 5. A PEM-only condition and uncoated surfaces were used as negative controls. Two conditions with 8 ng and 4 ng of exogenous FGF2 added at t = 0 days and t = 2 days were used as positive controls. The exogenous conditions had a PEM-only coating to serve as a direct comparison to the FGF2-eluting surfaces. The coated plates were sterilized under UV light for 10 min before starting culture.

Fibroblasts were initially expanded in complete growth medium (IMDM containing 10% FBS, 1% l-glutamate, and 1% penicillin streptomycin). Cells at passage 4 were seeded at a density of 5000 cells/cm^2^ (9500 cells/well) in individual 4-well plates for each condition and cultured in 600 µL of IMDM containing 1% FBS for 2 and 4 days at 37 °C and 5% CO_2_. At the specified time points, the 4-well plates were removed from the incubator. The media was aspirated and the cells were washed twice using 1× PBS with Mg^2+^ and Ca^2+^. The cells were fixed in 200 µL of 4% paraformaldehyde in PBS for 10 min, after which two more rinses in PBS were performed. The fixed cells were stored at 8 °C until staining.

To permeabilize the cells, 0.1% Triton X-100 in PBS was added to the culture wells for 5 min followed by two washes in PBS again. Cells were incubated in a 1 wt% BSA solution in PBS for 30 min to prevent non-specific binding of the fluorescent stains. After blocking, the wash step was repeated. The cell nuclei were stained using 300 µL of Hoechst solution diluted 1:3000 in PBS for 10 min under a foil cover. Phalloidin coupled with Alexa Fluor 488 was diluted to 2.5 vol.% before staining. 300 µL of solution was added per well and the stain was developed for 20 min under foil followed by two final washes with PBS.

The cells were imaged using a Leica SP8 Confocal Microscope. Cell counts based on the nuclei count and surface area values were obtained using ImageJ^50^.

### Statistical analysis

Statistical analysis was performed using a single factor analysis of variance (ANOVA) for all experiments. The Tukey Honest Significant Difference (HSD) method was used for post hoc analysis and was applied simultaneously for all conditions at their respective time points in an experimental data set.

## Supplementary Information

Supplementary Information

## References

[1] Tiwari G (2012). Drug delivery systems: An updated review. Int. J. Pharm. Investig..

[2] Boudou T, Crouzier T, Ren K, Blin G, Picart C (2010). Multiple functionalities of polyelectrolyte multilayer films: New biomedical applications. Adv. Mater..

[3] Ding I, Shendi DM, Rolle MW, Peterson AM (2018). Growth-factor-releasing polyelectrolyte multilayer films to control the cell culture environment. Langmuir.

[4] Peterson AM, Möhwald H, Shchukin DG (2012). pH-controlled release of proteins from polyelectrolyte-modified anodized titanium surfaces for implant applications. Biomacromol.

[5] Peterson AM, Pilz-Allen C, Kolesnikova T, Möhwald H, Shchukin D (2014). Growth factor release from polyelectrolyte-coated titanium for implant applications. ACS Appl. Mater. Interfaces.

[6] Guillot R (2013). The stability of BMP loaded polyelectrolyte multilayer coatings on titanium. Biomaterials.

[7] Ceccaldi C (2014). Evaluation of polyelectrolyte complex-based scaffolds for mesenchymal stem cell therapy in cardiac ischemia treatment. Acta Biomater..

[8] Calarco A (2010). Controlled delivery of the heparan sulfate/FGF-2 complex by a polyelectrolyte scaffold promotes maximal hMSC proliferation and differentiation. J. Cell. Biochem..

[9] Galderisi U (2013). Efficient cultivation of neural stem cells with controlled delivery of FGF-2. Stem Cell Res..

[10] She Z, Wang C, Li J, Sukhorukov GB, Antipina MN (2012). Encapsulation of basic fibroblast growth factor by polyelectrolyte multilayer microcapsules and its controlled release for enhancing cell proliferation. Biomacromol.

[11] Han, U. *et al.* Efficient encapsulation and sustained release of basic fibroblast growth factor in nanofilm: extension of the feeding cycle of human induced pluripotent stem cell culture. *ACS Appl. Mater. Interfaces* acsami.7b05519 (2017). 10.1021/acsami.7b05519 10.1021/acsami.7b05519 28686012

[12] Chu H, Gao J, Chen C-W, Huard J, Wang Y (2011). Injectable fibroblast growth factor-2 coacervate for persistent angiogenesis. Proc. Natl. Acad. Sci..

[13] Ding I, Walz JA, Mace CR, Peterson AM (2019). Early hMSC morphology and proliferation on model polyelectrolyte multilayers. Colloids Surf. B Biointerfaces.

[14] Sukhishvili SA, Kharlampieva E, Izumrudov V (2006). Where polyelectrolyte multilayers and polyelectrolyte complexes meet. Macromolecules.

[15] Salomäki M, Vinokurov IA, Kankare J (2005). Effect of temperature on the buildup of polyelectrolyte multilayers. Langmuir.

[16] She Z, Antipina MN, Li J, Sukhorukov GB (2010). Mechanism of protein release from polyelectrolyte multilayer. Biomacromol.

[17] Salvi C, Lyu X, Peterson AM (2016). Effect of assembly pH on polyelectrolyte multilayer surface properties and BMP-2 release. Biomacromol.

[18] Zhang H (2015). Effect of polyelectrolyte film stiffness on endothelial cells during endothelial-to-mesenchymal transition. Biomacromol.

[19] Garcia-Maya M (2006). Ligand concentration is a driver of divergent signaling and pleiotropic cellular responses to FGF. J. Cell. Physiol..

[20] Ahn, H.-J., Lee, W.-J., Kwack, K. & Kwon, Y. Do. FGF2 stimulates the proliferation of human mesenchymal stem cells through the transient activation of JNK signaling. *FEBS Lett.* **583**, 2922–2926 (2009). 10.1016/j.febslet.2009.07.056 19664626

[21] Page RL (2009). Induction of stem cell gene expression in adult human fibroblasts without transgenes. Cloning Stem Cells.

[22] Presta M (2005). Fibroblast growth factor/fibroblast growth factor receptor system in angiogenesis. Cytokine Growth Factor Rev..

[23] Narita A (2009). Effect of gelatin hydrogel incorporating fibroblast growth factor 2 on human meniscal cells in an organ culture model. Knee.

[24] Almodóvar J, Bacon S, Gogolski J, Kisiday JD, Kipper MJ (2010). Polysaccharide-based polyelectrolyte multilayer surface coatings can enhance mesenchymal stem cell response to adsorbed growth factors. Biomacromol.

[25] Crouzier T, Fourel L, Boudou T, Albigès-Rizo C, Picart C (2011). Presentation of BMP-2 from a soft biopolymeric film unveils its activity on cell adhesion and migration. Adv. Mater..

[26] Shah NJ (2011). Tunable dual growth factor delivery from polyelectrolyte multilayer films. Biomaterials.

[27] Müller S (2008). VEGF-functionalized polyelectrolyte multilayers as proangiogenic prosthetic coatings. Adv. Funct. Mater..

[28] Boura C (2005). Endothelial cell—interactions with polyelectrolyte multilayer films. Biomaterials.

[29] Dvorak P (2018). Computer-assisted engineering of hyperstable fibroblast growth factor 2. Biotechnol. Bioeng..

[30] Chaganti LK, Venkatakrishnan N, Bose K (2018). An efficient method for FITC labelling of proteins using tandem affinity purification. Biosci. Rep..

[31] Shiba T (2003). Modulation of mitogenic activity of fibroblast growth factors by inorganic polyphosphate. J. Biol. Chem..

[32] Kurien B, Scofield R (2006). Western blotting. Methods.

[33] MacPhee DJ (2010). Methodological considerations for improving Western blot analysis. J. Pharmacol. Toxicol. Methods.

[34] Sakamoto S (2018). Enzyme-linked immunosorbent assay for the quantitative/qualitative analysis of plant secondary metabolites. J. Nat. Med..

[35] Sanchez-Ruiz JM (2010). Protein kinetic stability. Biophys. Chem..

[36] Shiratori SS, Rubner MF (2000). pH-dependent thickness behavior of sequentially adsorbed layers of weak polyelectrolytes. Macromolecules.

[37] Sui Z, Salloum D, Schlenoff JB (2003). Effect of molecular weight on the construction of polyelectrolyte multilayers: Stripping versus sticking. Langmuir.

[38] Siepmann J, Peppas NA (2012). Modeling of drug release from delivery systems based on hydroxypropyl methylcellulose (HPMC). Adv. Drug Deliv. Rev..

[39] Peppas, N. A. & Korsmeyer, R. Dynamically swelling hydrogels in controlled release applications. in *Hydrogels in Medicine and Pharmacy, Vol. 3* 109–135 (CRC Press, 1986).

[40] Kole D, Ambady S, Page RL, Dominko T (2014). Maintenance of multipotency in human dermal fibroblasts treated with Xenopus laevis egg extract requires exogenous fibroblast growth factor-2. Cell. Reprogram..

[41] Grella, A., Kole, D., Holmes, W. & Dominko, T. FGF2 Overrides TGF b 1-driven integrin ITGA11 expression in human dermal fibroblasts. **1008**, 1000–1008 (2016). 10.1002/jcb.25386 26403263

[42] Whitney ML, Otto KG, Blau CA, Reinecke H, Murry CE (2001). Control of myoblast proliferation with a synthetic ligand. J. Biol. Chem..

[43] Clegg CH, Linkhart TA, Olwin BB, Hauschka SD (1987). Growth factor control of skeletal muscle differentiation: Commitment to terminal differentiation occurs in G1phase and is repressed by fibroblast growth factor. J. Cell Biol..

[44] Dolivo DM, Larson SA, Dominko T (2017). FGF2-mediated attenuation of myofibroblast activation is modulated by distinct MAPK signaling pathways in human dermal fibroblasts. J. Dermatol. Sci..

[45] Kole D (2017). High molecular weight FGF2 isoforms demonstrate canonical receptor-mediated activity and support human embryonic stem cell self-renewal. Stem Cell Res..

[46] Tew LS, Ching JY, Ngalim SH, Khung YL (2018). Driving mesenchymal stem cell differentiation from self-assembled monolayers. RSC Adv..

[47] Stephenson, M. & Grayson, W. Recent advances in bioreactors for cell-based therapies. *F1000Research* **7**, 517 (2018). 10.12688/f1000research.12533.1 PMC5931275 29770207

[48] Edvardsson, M. Why are overtones important in QCM? *Biolin Sci. *(2018).

[49] Vogt BD, Lin EK, Wu W, White CC (2004). Effect of film thickness on the validity of the sauerbrey equation for hydrated polyelectrolyte films. J. Phys. Chem. B.

[50] Schneider CA, Rasband WS, Eliceiri KW (2012). NIH image to ImageJ: 25 years of image analysis. Nat. Methods.

