# Supplementary material for: Half-life modeling of basic fibroblast growth factor released from growth factor-eluting polyelectrolyte multilayers
Source: Sci Rep. 2021 May 7;11:9808. doi: 10.1038/s41598-021-89229-w (PMC8105364; doi:10.1038/s41598-021-89229-w)
Supplement: Supplementary file 1 — Supplementary Information [file 41598_2021_89229_MOESM1_ESM.docx]

**Supporting Information** for “Half-Life Modeling of Basic Fibroblast Growth Factor released from Growth Factor-Eluting Polyelectrolyte Multilayers”

Ivan Ding^a^, Amy M. Peterson*^b^

^a^Department of Chemical Engineering, University of Massachusetts Lowell, One University Ave., Lowell, MA, 01854

^b^Department of Plastics Engineering, University of Massachusetts Lowell, One University Ave., Lowell, MA, 01854

*amy_peterson@uml.edu

**Contents**

**Figure S.1** Overall recovered FGF2 and percentage release of FGF2 over time

**Figure S.2** Cumulative FGF2 release plotted against the natural log of time

**Figure S.3** Original release data adjusted with half-life value and Power Law fit

**Figure S.4** Scaled Power Law data compared to actual cell culture concentration

**Table S.1** Half-life values obtained by fitting entire time range

**Table S.2** Power law fit constants for all modeled data

**Equation S.1** Higuchi Equation

**Figure S.5** FGF2 cell culture concentration using Higuchi data

**Figures S.6- S.17** Large-scale representative cell images


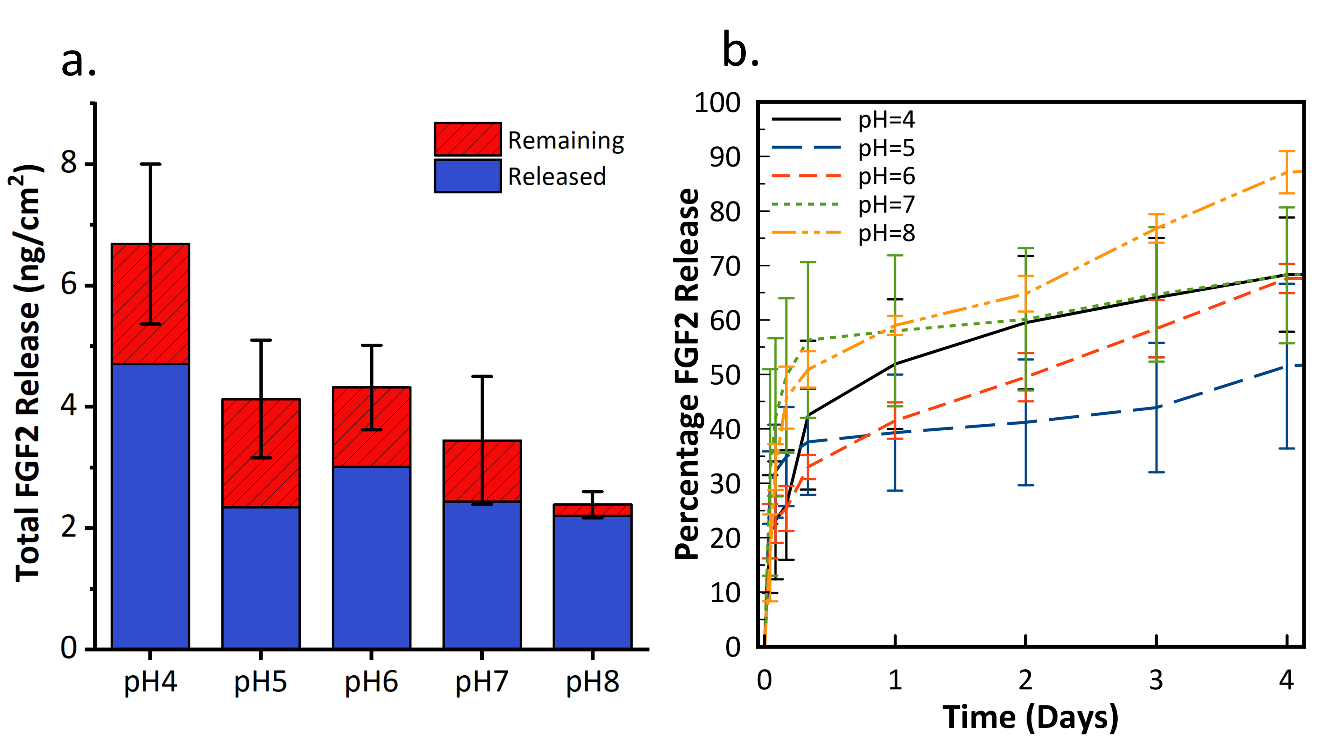


**Figure S.1: a.** Overall FGF2 release from PEM, plus remaining FGF2 recovered from acid/base wash step. Released FGF2 corresponds to FGF2 release seen in Figure 2 in the body of the publication. **b.** Percentage FGF2 release based on overall FGF2 recovered. Error bars represent standard deviation (n=5)


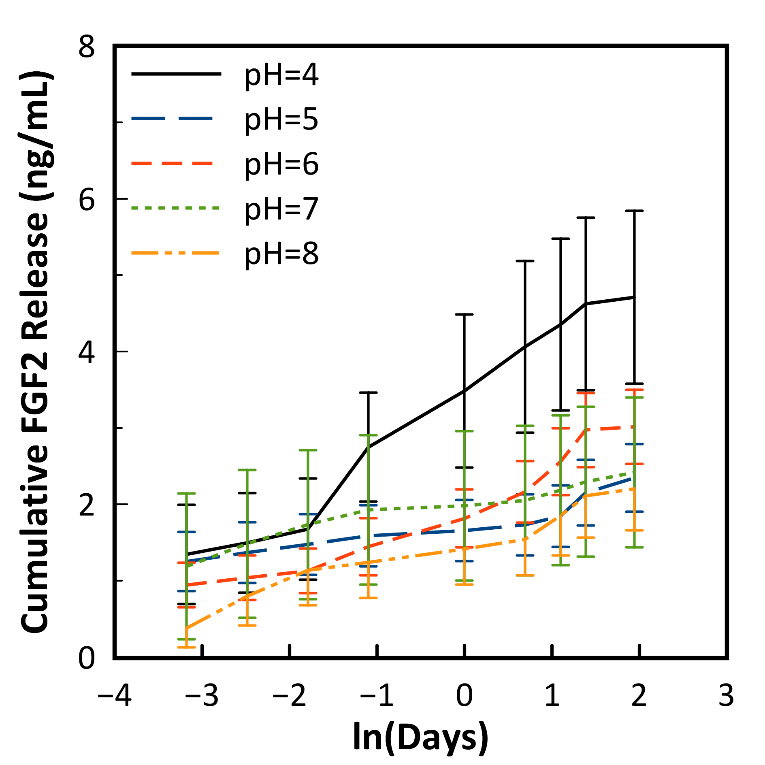


**Figure S.2:** Cumulative FGF2 release as seen in Figure 2 of the body of the publication plotted against the natural log of time to improve clarity of initial time points. Error bars represent standard deviation (n=5)

**Table S.1:** Half-life values obtained using data in Figure 1 in the body of the publication, fitting all data points including the first 8-hour period to the first order rate law. The half-life values are lower than the reported values in the body of the publication due to the non-equilibrium state during the first few hours.

| **Condition** | **Half Life (hr)** | **Std. Dev. (hr)** | **R^2^ Value** |
| --- | --- | --- | --- |
| PBS (500 ng/mL) | 21.64 | 2.26 | 0.91 |
| PBS (250 ng/mL) | 22.84 | 4.10 | 0.88 |
| DMEM (500 ng/mL) | 22.86 | 3.67 | 0.86 |
| DMEM (250 ng/mL) | 22.61 | 4.15 | 0.94 |

**Table S.2:** Power Law fit parameters for PEMs formed from pH=4 to pH=8. Values assume a 1.9 cm^2^ substrate surface area and 600 μL of cell culture media.

| **Variable** | **pH=4** | **pH=5** | **pH=6** | **pH=7** | **pH=8** |
| --- | --- | --- | --- | --- | --- |
| **K** | 4.14 | 3.88 | 2.70 | 4.26 | 1.79 |
| **n** | 0.308 | 0.134 | 0.286 | 0.141 | 0.315 |


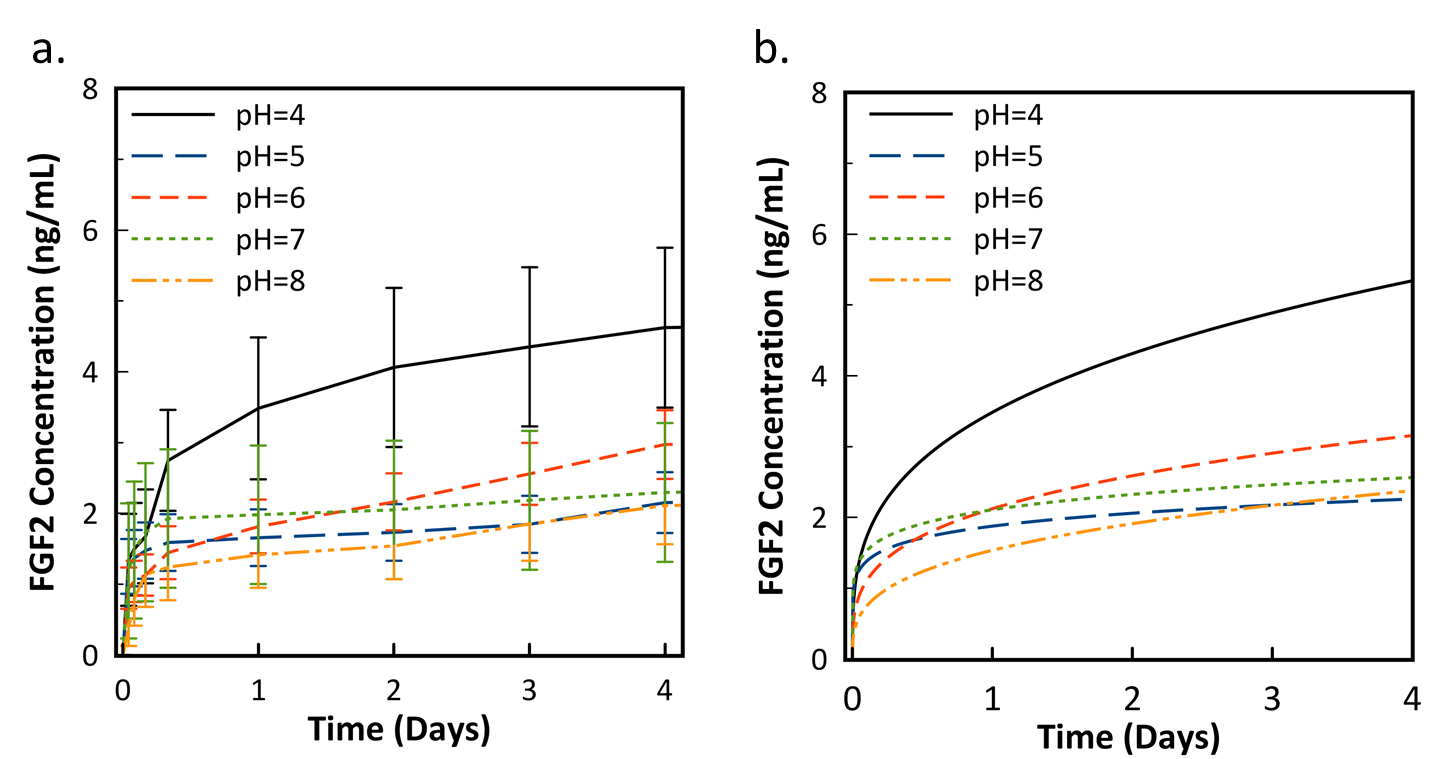


**Figure S.3: a.** Cumulative FGF2 release over 4 days from FGF2-(PMAA/PLH)5 PEMs, as seen in Figure 2 in the body of the manuscript. Error bars represent standard deviation (n=5) **b.** Power Law fit of experimental data with degradation during release factored into the calculation. Factoring in half-life results in a higher predicted release more notable at the daily time steps. Error bars represent standard deviation (n=5).


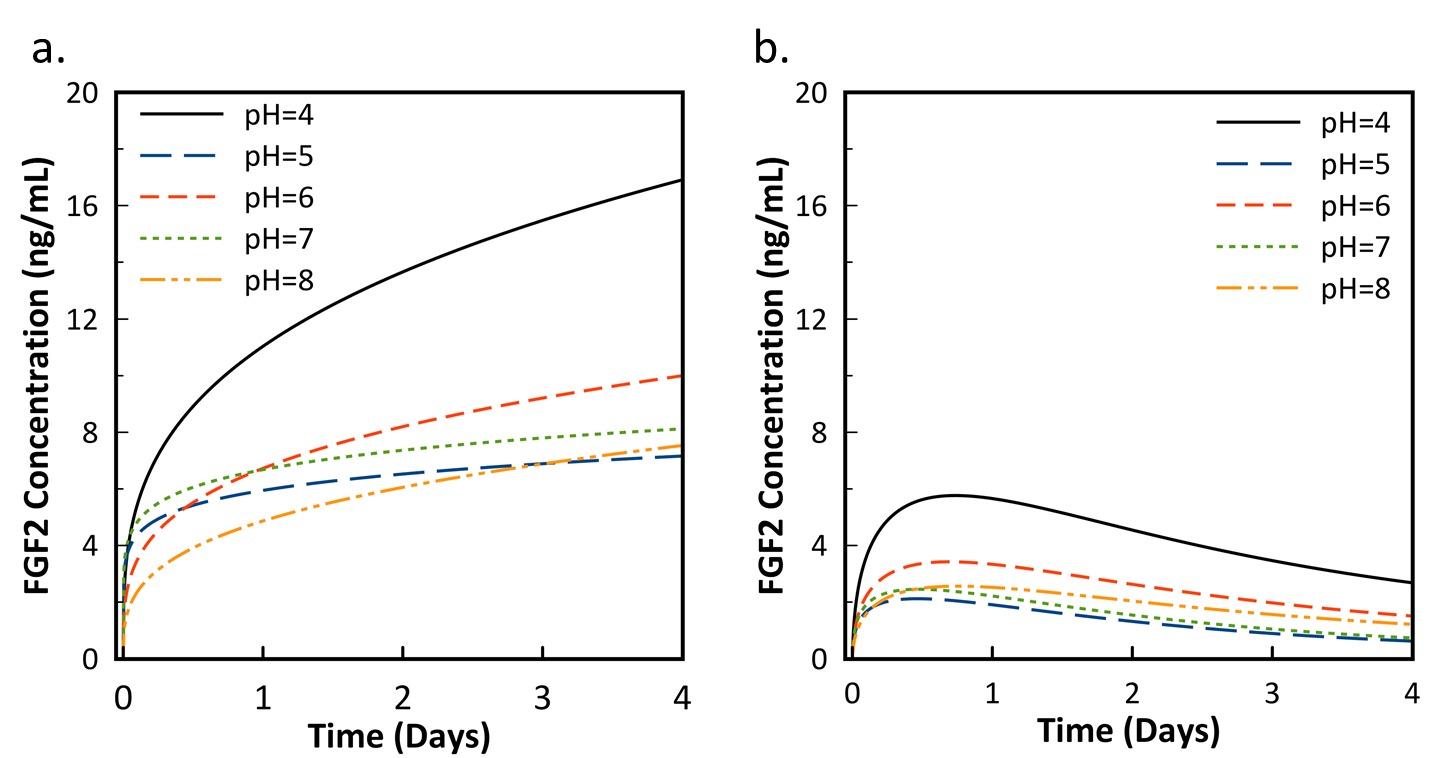


**Figure S.4: a.** Power Law fit of experimental data adjusted to dimensions and volumes of a 1.9 cm^2^ well plate. Data assumes cumulative release without any degradation in FGF2 **b.** Release curve on same scale (data identical to Figure 4a in main text of paper) assuming degradation of FGF2 over time.

**Equation S.1**: Higuchi Equation. Serves as a general drug release model and was the first attempted model. The power law is more appropriate due to swelling of the PEM. M_t_ is the cumulative release up to a specific time t, M_∞_ is the total amount of drug released at an infinite time period and K is a constant representing diffusivity, drug solubility and release area.

$$\frac{M_{t}}{M_{\infty}}=K\sqrt{t}$$

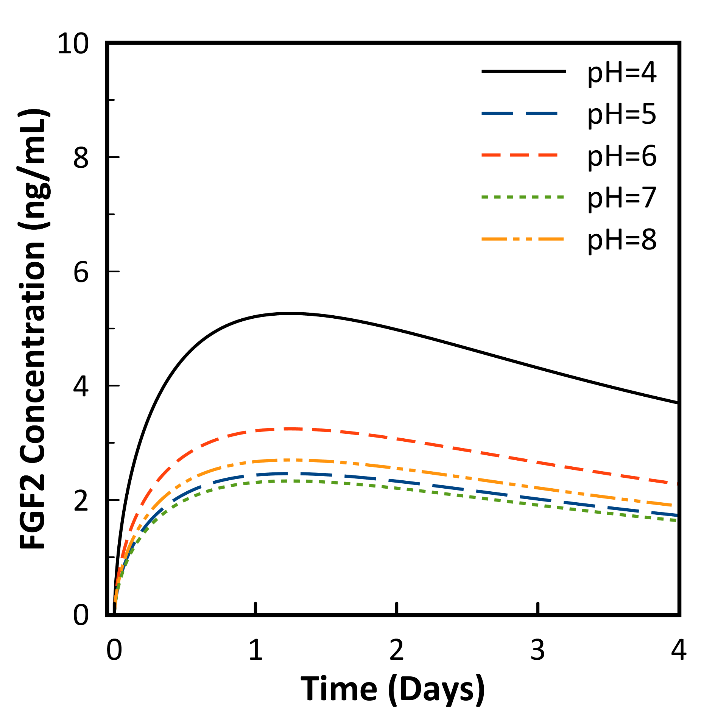


**Figure S.5:** Release curve data fit to the Higuchi equation. As the Higuchi equation less effectively predicts burst release, peak concentration predicted is lower, but concentration range is also reduced.


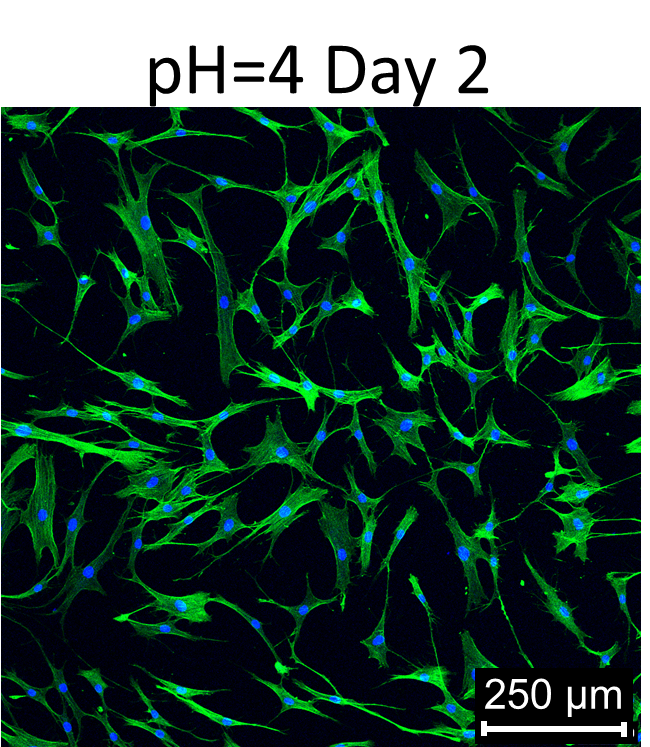


**Figure S.6:** Large-scale image of cells grown in the pH=4 condition at day 2 seen in Figure 5 in the main body of the publication. Cells stained with Hoechst 33342 (blue) and phalloidin coupled with Alexa Fluor 488 (green)


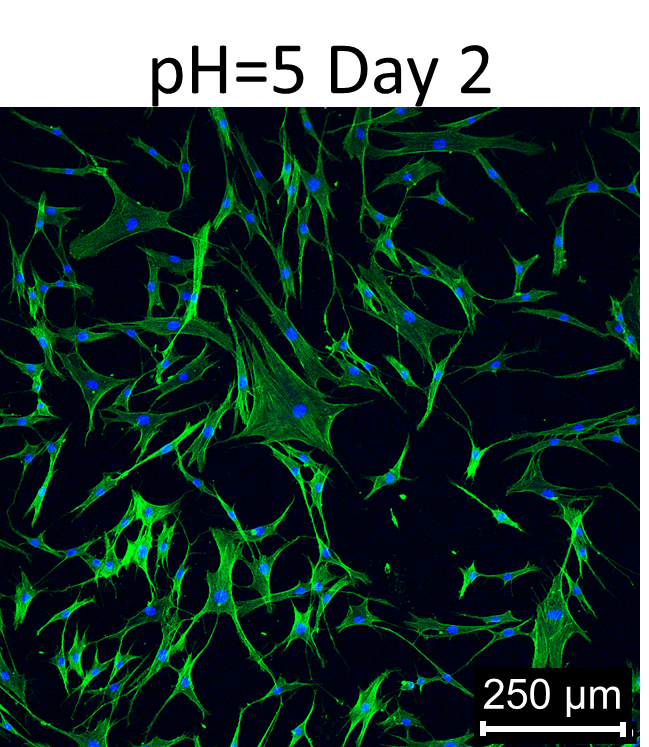


**Figure S.7:** Large-scale image of cells grown in the pH=5 condition at day 2 seen in Figure 5 in the main body of the publication. Cells stained with Hoechst 33342 (blue) and phalloidin coupled with Alexa Fluor 488 (green)


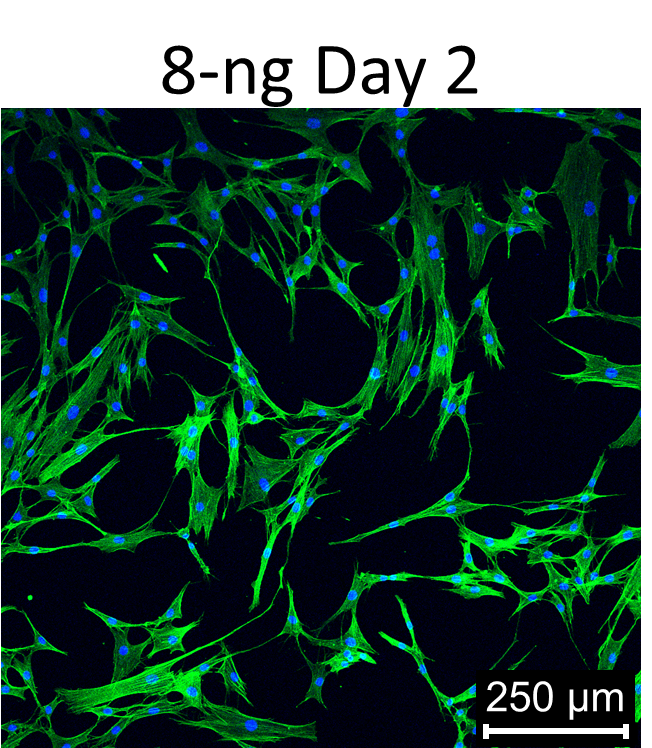


**Figure S.8:** Large-scale image of cells grown in the 8-ng exogenous condition at day 2 seen in Figure 5 in the main body of the publication. Cells stained with Hoechst 33342 (blue) and phalloidin coupled with Alexa Fluor 488 (green)


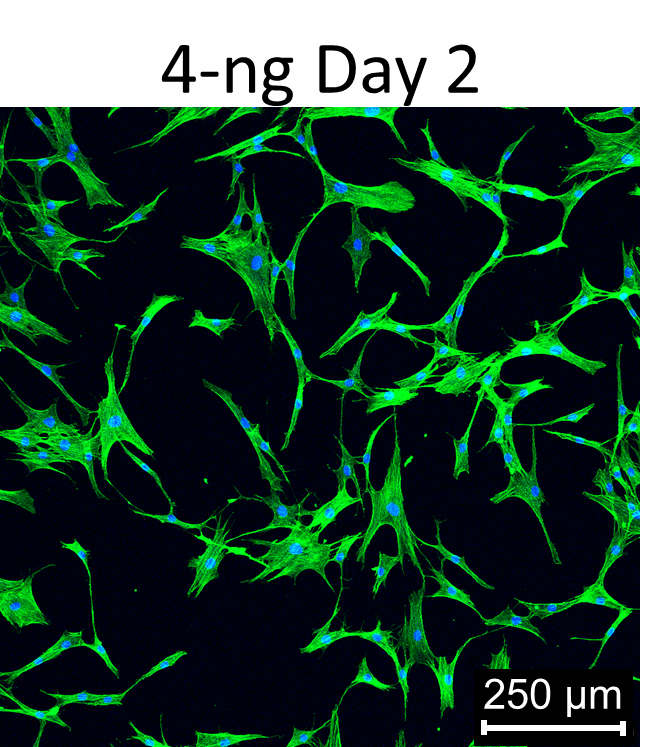


**Figure S.9:** Large-scale image of cells grown in the 4-ng exogenous condition at day 2 seen in Figure 5 in the main body of the publication. Cells stained with Hoechst 33342 (blue) and phalloidin coupled with Alexa Fluor 488 (green)


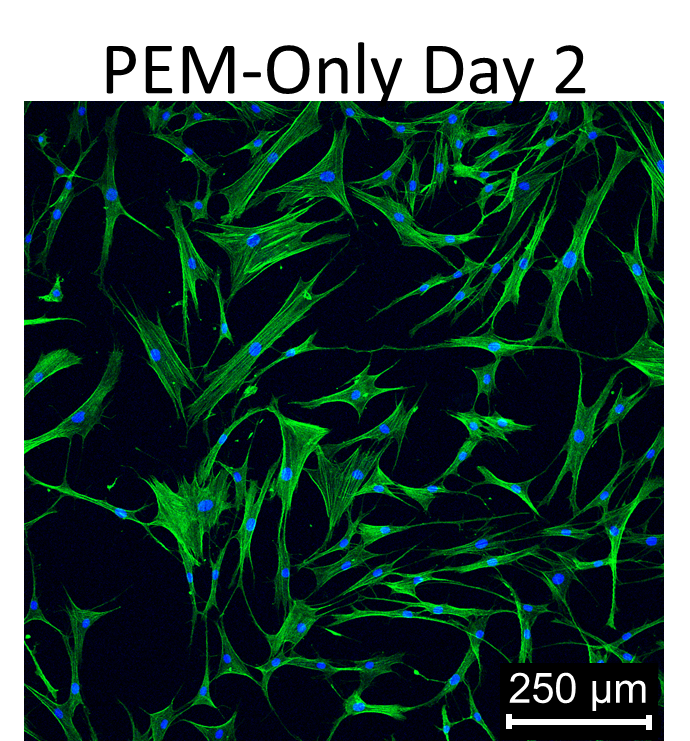


**Figure S.10:** Large-scale image of cells grown in the PEM=only condition (no FGF2 release) at day 2 seen in Figure 5 in the main body of the publication. Cells stained with Hoechst 33342 (blue) and phalloidin coupled with Alexa Fluor 488 (green)


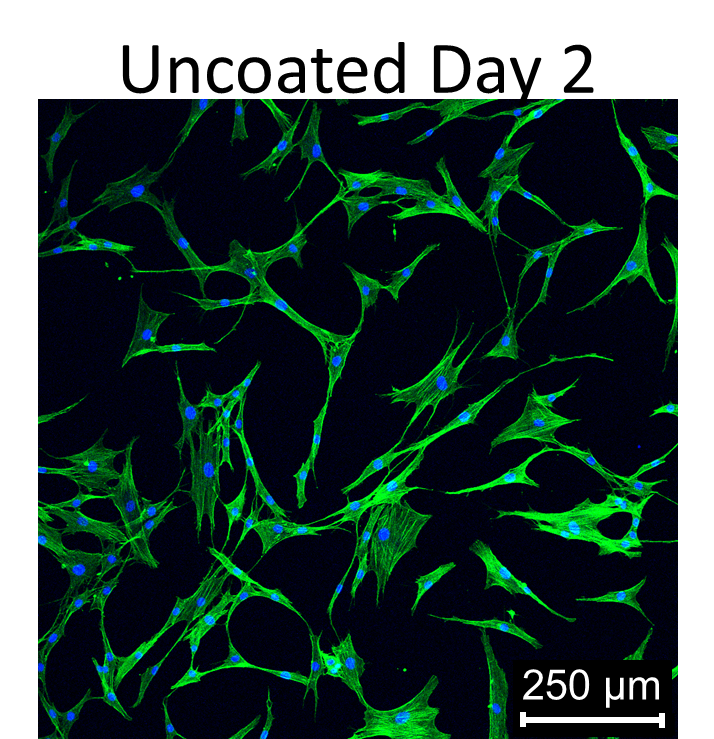


**Figure S.11:** Large-scale image of cells grown in the uncoated condition at day 2 seen in Figure 5 in the main body of the publication. Cells stained with Hoechst 33342 (blue) and phalloidin coupled with Alexa Fluor 488 (green)


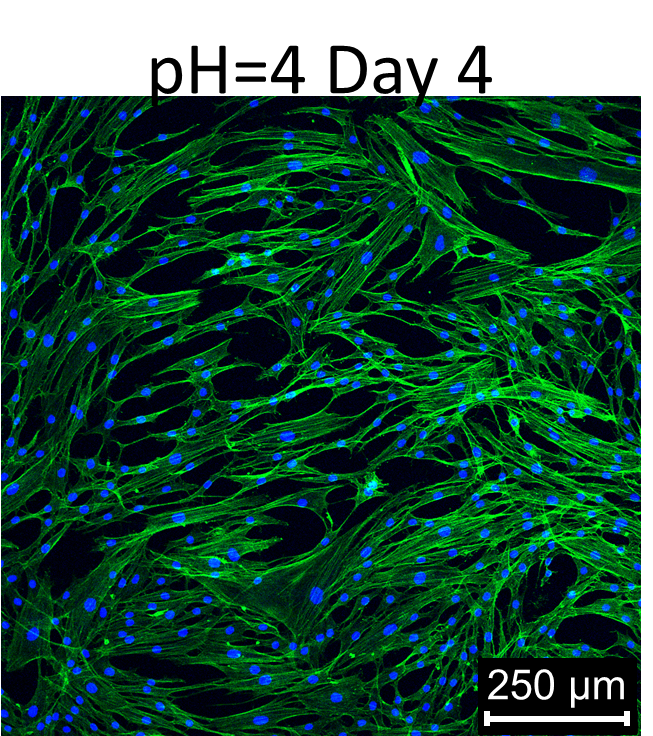


**Figure S.12:** Large-scale image of cells grown in the pH=4 condition at day 4 seen in Figure 5 in the main body of the publication. Cells stained with Hoechst 33342 (blue) and phalloidin coupled with Alexa Fluor 488 (green)


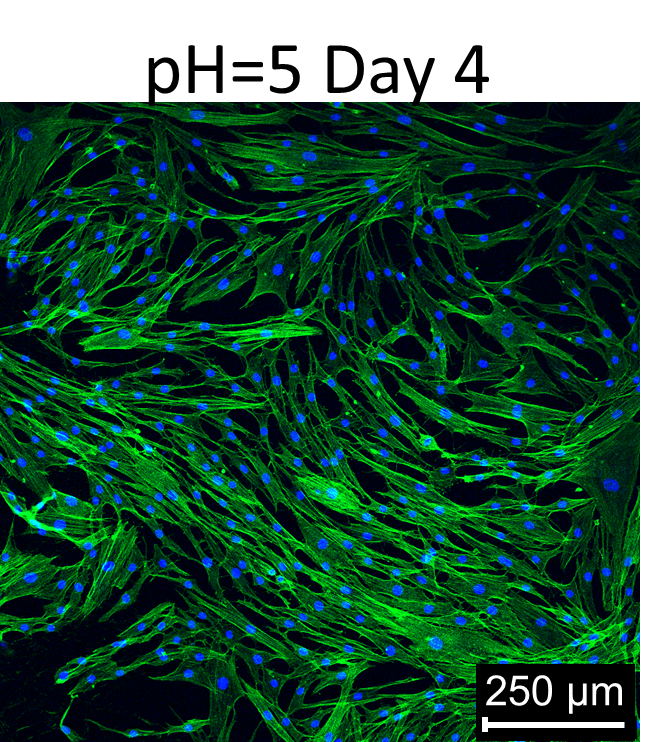


**Figure S.13:** Large-scale image of cells grown in the pH=4 condition at day 4 seen in Figure 5 in the main body of the publication. Cells stained with Hoechst 33342 (blue) and phalloidin coupled with Alexa Fluor 488 (green)


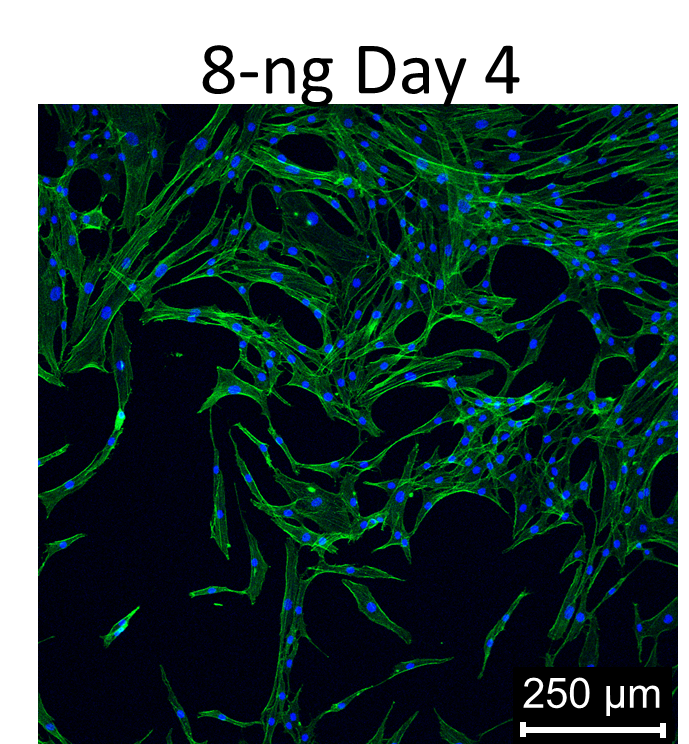


**Figure S.14:** Large-scale image of cells grown in the 8-ng exogenous condition at day 4 seen in Figure 5 in the main body of the publication. Cells stained with Hoechst 33342 (blue) and phalloidin coupled with Alexa Fluor 488 (green)


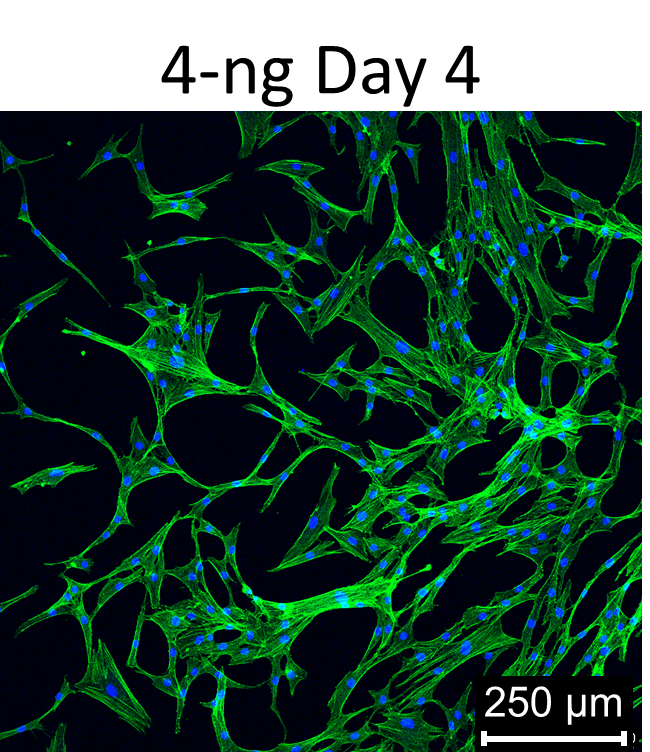


**Figure S.15:** Large-scale image of cells grown in the 4-ng exogenous condition at day 4 seen in Figure 5 in the main body of the publication. Cells stained with Hoechst 33342 (blue) and phalloidin coupled with Alexa Fluor 488 (green)


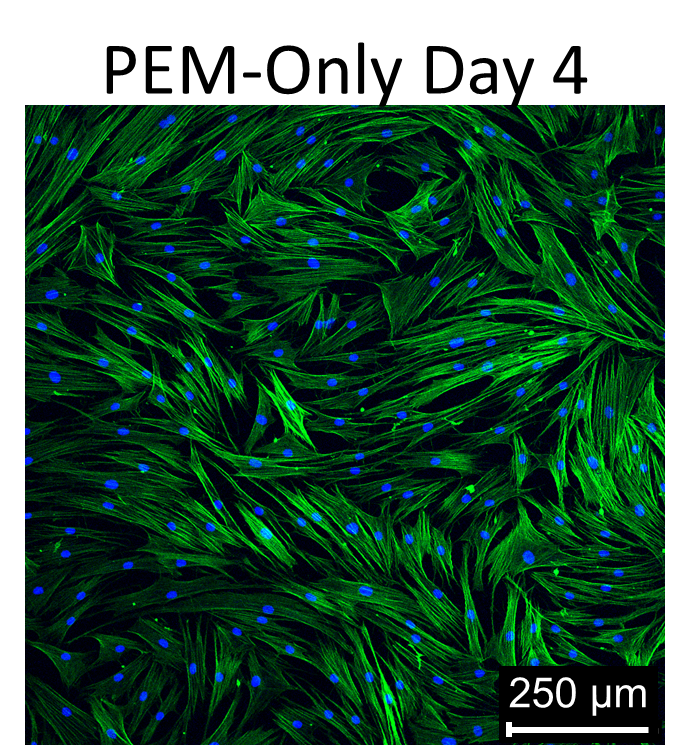


**Figure S.16:** Large-scale image of cells grown in the PEM=only condition (no FGF2 release) at day 4 seen in Figure 5 in the main body of the publication. Cells stained with Hoechst 33342 (blue) and phalloidin coupled with Alexa Fluor 488 (green)


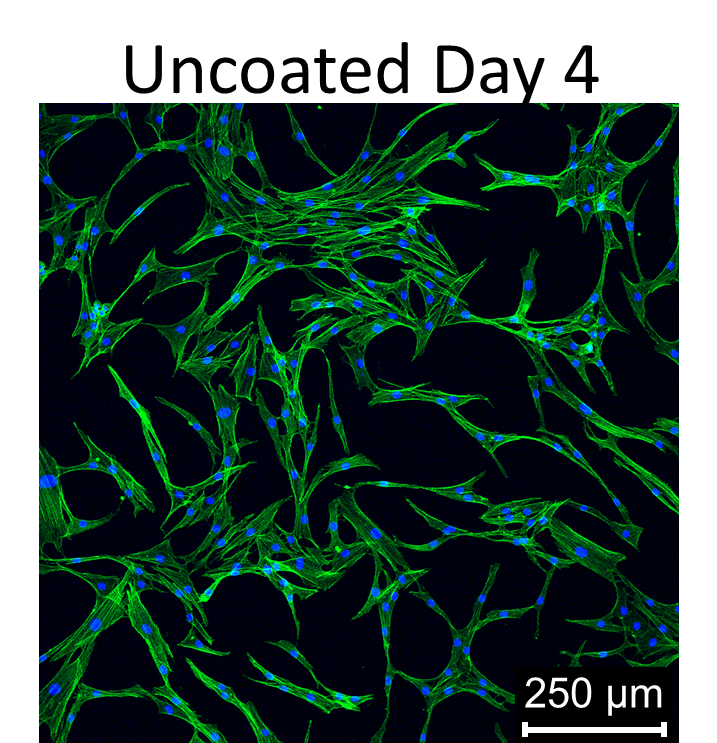


**Figure S.17:** Large-scale image of cells grown in the uncoated condition at day 4 seen in Figure 5 in the main body of the publication. Cells stained with Hoechst 33342 (blue) and phalloidin coupled with Alexa Fluor 488 (green)
